# Molecular Mechanisms of Melatonin Protection from Gastric Mucosal Apoptotic Injury in Experimental Burns

**DOI:** 10.3390/molecules23040749

**Published:** 2018-03-24

**Authors:** Minka Hristova, Maria Tzaneva, Ganka Bekyarova, Dariya Chivchibashi, Nadezhda Stefanova, Yoana Kiselova-Kaneva

**Affiliations:** 1 Department of Physiology and Pathophysiology, Medical University of Varna, Varna 9002, Bulgaria; ganka.bekyarova@gmail.com (G.B.); dariya.chi@gmail.com (D.C.); 2 Department of Preclinical and Clinical Sciences, Medical University of Varna, Varna 9002, Bulgaria; mtzaneva@hotmail.com; 3 Department of General and Clinical Pathology, Forensic Science and Deontology, Medical University of Varna, Varna 9010, Bulgaria; nadezhda_stefanova@yahoo.com; 4 Department of Biochemistry, Molecular Medicine and Nutrigenomics, Medical University of Varna; Varna 9002, Bulgaria; yoana.kiselova@mu-varna.bg

**Keywords:** melatonin, burns, gastric injury, 4-HNE, Bcl-2, Bax, Nrf2

## Abstract

Melatonin, a basic secretory pineal gland product, is a nontoxic, multifunctional molecule. It has antioxidant and anti-apoptotic activities and protects tissues from injury. The objective of the present study was to determine the molecular mechanism of melatonin anti-apoptotic effect on gastric injury in a rat burn model. We hypothesized that melatonin gastric protection may be related to the activation of transcription erythroid 2-related factor 2 (Nrf2). Using a 30% total body surface area (TBSA) rat burn model, melatonin (10 mg/kg, i.p.) was injected immediately and 12 h after thermal skin injury. Via light immunohistochemistry, we determined the tissue level of 4-hydroxy-2-nonenal (4-HNE) as a marker of lipid peroxidation, Bcl-2 and Bax as apoptosis-related proteins, and Nrf2. Results are presented as medians (interquartile range (IQR)). Thermal trauma in burned animals, compared with the controls, increased the expression of pro-apoptotic Bax protein (1.37 (0.94–1.47)), decreased anti-apoptotic Bcl-2 protein (1.16 (1.06–1.23), *p* < 0.001) in epithelial cells, and elevated Bax/Bcl-2 ratios (*p* < 0.05). Tissue 4-HNE and Nrf2 levels were increased following severe burns (1.55 (0.98–1.61) and 1.16 (1.01–1.25), *p* < 0.05, respectively). Melatonin significantly decreased 4-HNE (0.87 (0.74–0.96), *p* < 0.01) and upregulated Nrf2 (1.55 (1.52–1.65), *p* < 0.001) levels. It also augmented Bax (1.68 (1.5–1.8), *p* < 0.001) and Bcl-2 expressions (1.96 (1.89–2.01), *p* < 0.0001), but reduced Bax/Bcl-2 ratios (*p* < 0.05). Our results suggest that experimental thermal trauma induces oxidative gastric mucosal injury. Melatonin manifests a gastroprotective effect through Nrf2 activation, lipid peroxidation attenuation, and Bax/Bcl-2 ratio modification as well.

## 1. Introduction

Severe thermal trauma induces stomach damage and dysfunction. Pathogenesis of gastric mucosal injury is complex and has not yet been completely clarified. Local skin insult leads to generalized response—inflammation and oxidative stress [1,2]. Ischemia and reperfusion of the abdominal organs leads to neutrophil infiltration, overproduction of both reactive oxygen species (ROS) and reactive nitrogen species (RNS), and increased cytokines secretion. ROS/RNS and lipid peroxidation is an important mechanism for distant organ injury in burns. The 4-hydroxy-2-nonenal (4-HNE), one of the major lipid peroxidation products, is one of the toxic markers of mucosa injuring by ROS. There is increased 4-HNE level in oxidative gastric injury induced by ischemia reperfusion (I/R), ethanol, water immersion restraint stress, and non-steroidal anti-inflammatory drugs (NSAIDs) [3,4]. The 4-HNE induces apoptosis in a variety of cells [5,6]. There is, however, no evidence of its role in gastric mucosal apoptosis pathogenesis after thermal trauma.

Apoptosis (programmed cell death) is a genetically determined energy-dependent process and homeostatic mechanism, which maintains cell populations in tissues. Mechanisms of apoptosis are complex and involve a consistent cascade of processes and molecular interactions. There are two main apoptotic pathways: the extrinsic, or “death receptor,” pathway and the intrinsic mitochondrial pathway [7]. Death receptors are members of the tumor necrosis factor (TNF) receptor gene superfamily. The intrinsic pathway is usually activated under stress conditions, including DNA damage and oxidative stress. The regulators of this pathway are the Bcl-2 family proteins; anti-apoptotic (such as Bcl-2 and Bcl-xl) and pro-apoptotic (such as Bax, Bak, and Bad) [8]. The cell response (survival or death) to apoptopic stimulus depends on the balance between pro-apoptotic and anti-apoptotic Bcl-2 proteins [9].

The 4-HNE, as electrophile, can act as a signal molecule and increases transcriptional activity of nuclear factors such as NF-κB, Nrf2, and AP.

Nuclear factor (erythroid-derived 2)-like 2 (Nrf2) is an important cytoprotective transcription factor [10]. It is known that Nrf2 plays a key role in the antioxidant protection of the organism and in the redox signaling pathways. Under conditions of oxidative stress and increased lipid peroxidation (4-HNE), Nrf2 rapidly translocates into the nucleus [11]. The Nrf2 target genes encode detoxification enzymes and cytoprotective proteins associated with antioxidant defense and apoptosis [12].

Melatonin (N-acetyl-5-methoxytryptamine) is the primary neurohormone secreted in the pineal gland. It has pronounced pleiotropic biological activities, e.g. endocrine, autocrine, and paracrine [13]. The antioxidation is one of its main peripheral functions [14]. Melatonin reduces lipid peroxidation and modulates balance between pro-apoptotic and anti-apoptotic Bcl-2 proteins, depending on cell type (damaged cell or neoplastic cell) [15,16]. Moreover, melatonin also has a potent protective effect on gastric mucosal injury induced by NSAIDs, stress, and I/R [17,18]. It regulates mitochondrial homeostasis [19]. Melatonin modulates the expression of Bcl-2 family proteins and restricts the burn-induced liver damage [20].

The aim of the current study was to investigate the protective effect of melatonin, a potent antioxidant with pleiotropic activity, in burn-induced gastric mucosal injury by measuring a marker of lipid oxidative damage (4-HNE) and expression of apoptosis-related proteins Bcl-2 and Bax in rats. We hypothesize that the molecular mechanisms of antiapoptotic effects of melatonin are mediated by transcription factor Nrf2 activation.

## 2. Results

### 2.1. The Effect of Melatonin on 4-HNE Expression in Gastric Mucosa

**Figure 1:**
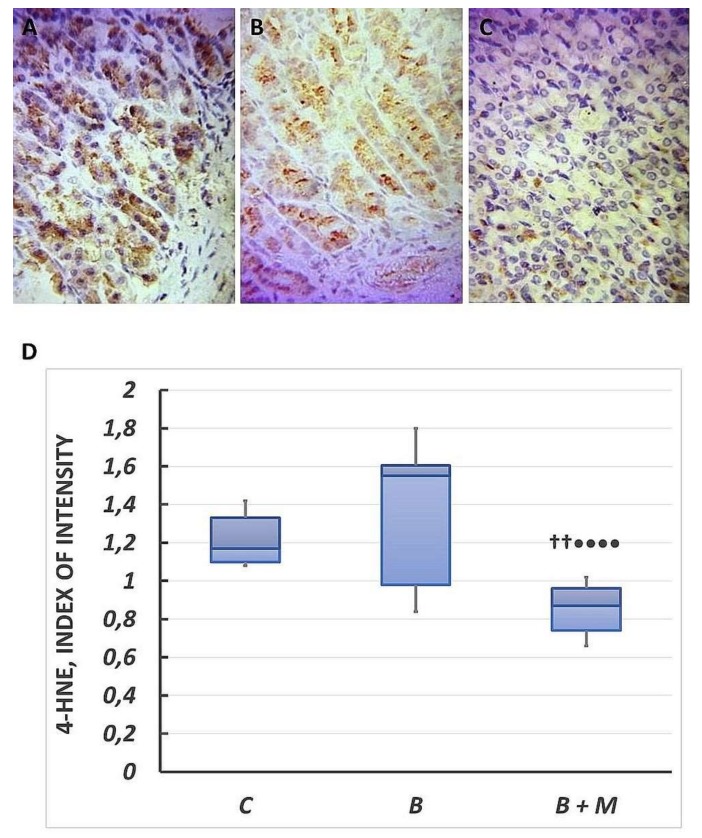
Melatonin effect on 4-HNE expression. Immunohistochemical 4-HNE detection in gastric mucosa. Controls (**A**); burned rats (**B**); burned melatonin-treated rats (**C**). The antigen site appears as a brown color. Representative images. Original magnification, 400×. Score index of 4-HNE positive immunostained cells (**D**). Results are given as box plot, with median, 25th- and 75th-percentile values, and min and max values. ^††^ *p* < 0.01 vs. burned, non-treated group; ^••••^ *p* < 0.0001 vs. control group. Controls (C); burned rats (B); burned melatonin-treated rats (B + M).

Immunohistochemistry showed 4-HNE cytoplasmic expression in epithelial cells in the control group. It was mainly observed in 1/3 of the basal region of the gastric glands where reaction intensity appeared to be weak to moderate. The cytoplasmic content was 1.17 (1.1–1.33) (Figure 1A). In the burned group, the 4-HNE-positive cells were also detected in the basal portion of gastric glands showing weak to moderate reaction intensity, while the epithelial cells from the upper portion of gastric glands remain negative (Figure 1B). The cytoplasmic expression was 1.55 (0.98–1.61) and increased by 32% when compared to the control group. In the burned and melatonin-treated group, 4-HNE cytoplasmic expression was strongly reduced to 0.87 (0.74–0.96), *p* < 0.01 (Figure 1C).

### 2.2. The Melatonin Effect on Bax Protein Expression in Gastric Mucosa

**Figure 2:**
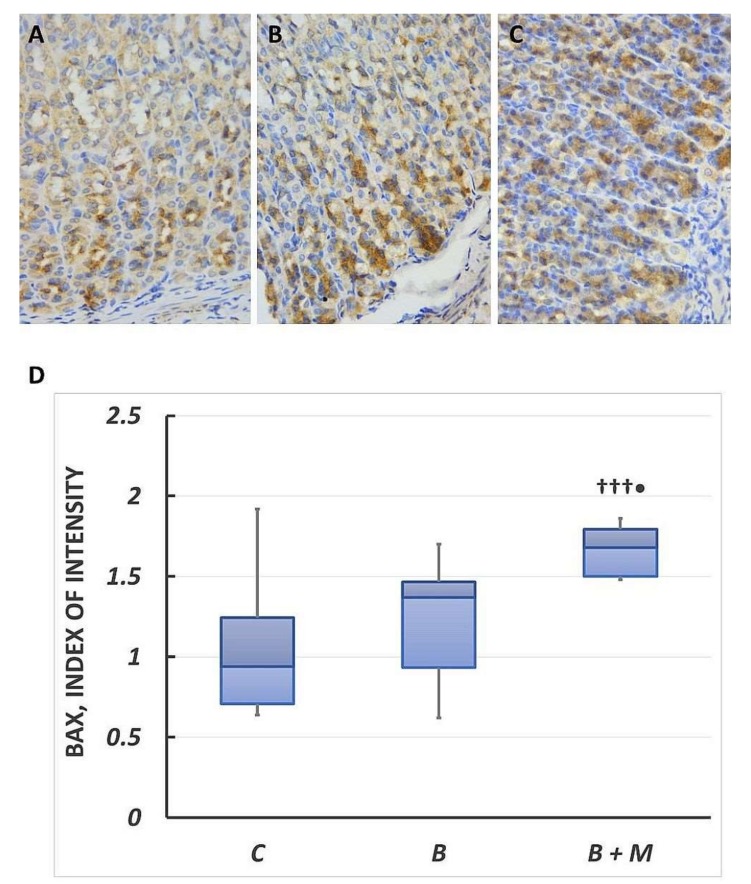
Melatonin effect on Bax protein expression. Immunohistochemical detection of Bax in gastric mucosa. Controls (**A**); burned rats (**B**); burned melatonin-treated rats (**C**). The antigen site appears as a brown color. Representative images. Original magnification, 400×. Score index of Bax positive immunostained cells (**D**). Results are given as box plot, with median, 25th- and 75th-percentile values, and min and max values. ^†††^ *p* < 0.001 vs. burned, non-treated group; ^•^ *p* < 0.05 vs. control group. Controls (C); burned rats (B); burned melatonin-treated rats (B + M).

The results of immunohistochemistry in the control group showed positive Bax expression in the cytoplasm of epithelial cells with predominance in basal portion of gastric glands (0.94 (0.71–1.25)) (Figure 2A). The burned group showed stronger expression of Bax (1.37 (0.94–1.47)) and it was higher by 46% compared to the control group (Figure 2B). In the burned melatonin-treated group, cytoplasmic Bax protein expression was detected in most epithelial cells in the gastric mucosa (Figure 2C). The reaction intensity was stronger (1.68 (1.5–1.8)) and significantly higher compared to both burned and control groups (23%, *p* < 0.001 and 79%, *p* < 0.05, respectively).

### 2.3. The Melatonin Effect on Bcl-2 Protein Expression in Gastric Mucosa

**Figure 3:**
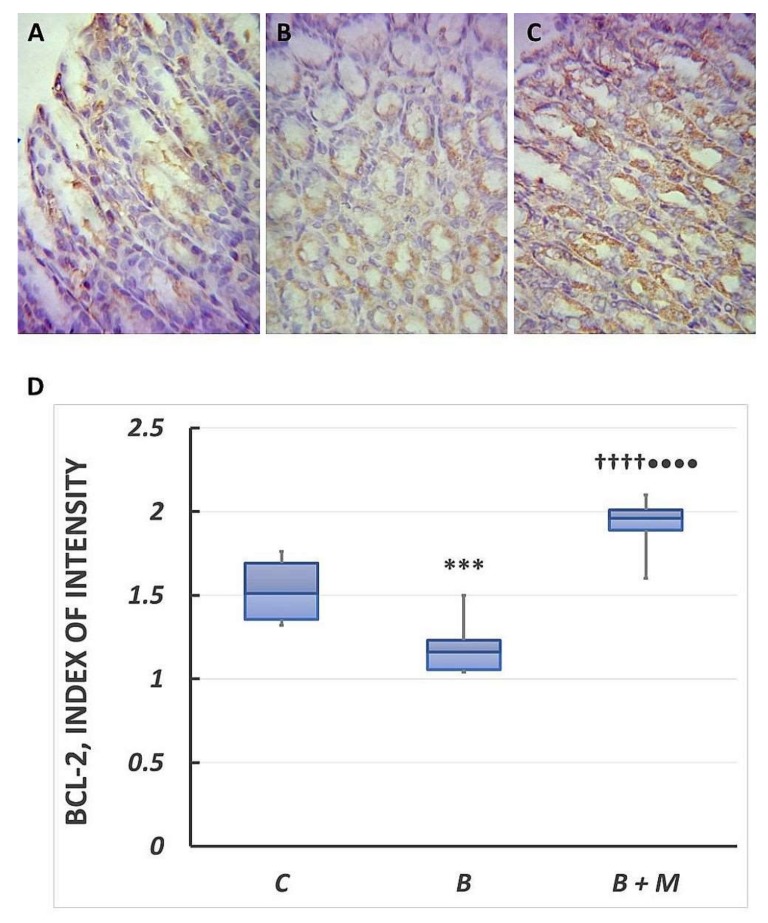
Melatonin effect on Bcl-2 expression. Immunohistochemical detection of Bcl-2 in gastric mucosa. Controls (**A**); burned rats (**B**); burned melatonin-treated rats (**C**). The antigen site appears as a brown color. Representative images. Original magnification, 400×. Score index of Bcl-2 positive immunostained cells (**D**). Results are given as box plot, with median, 25th- and 75th-percentile values, and min and max values. *** *p* < 0.001 vs. control group; ^††††^ *p* < 0.0001 vs. burned, non-treated group; ^••••^ *p* < 0.0001 vs. control group. Controls (C); burned rats (B); burned melatonin-treated rats (B + M).

Bcl-2 cytoplasmic expression observed predominantly in the basal parts of the gastric glands in the control group (Figure 3A). The content was 1.51 (1.36–1.69). In contrast, the Bcl-2 expression was more diffuse in the burned group. Furthermore, it was detected in both the nucleus and the cytoplasm of gastric epithelial cells. The reaction intensity was 1.16 (1.06–1.23) and statistically significantly lower by 23% (*p* < 0.001) compared to the control group (Figure 3B). In the melatonin-treated group, cytoplasmic Bcl-2 protein expression was detected in almost all the cells of the gastric mucosa (Figure 3B). Its reaction intensity was 1.96 (1.89–2.01). The Bcl-2 response was higher compared to both groups (69% vs. burned group (*p* < 0.0001); 30% vs. control group (*p* < 0.0001)).

### 2.4. Changes in Bax/Bcl-2 Ratio in Gastric Mucosa Following Burn Trauma

**Figure 4:**
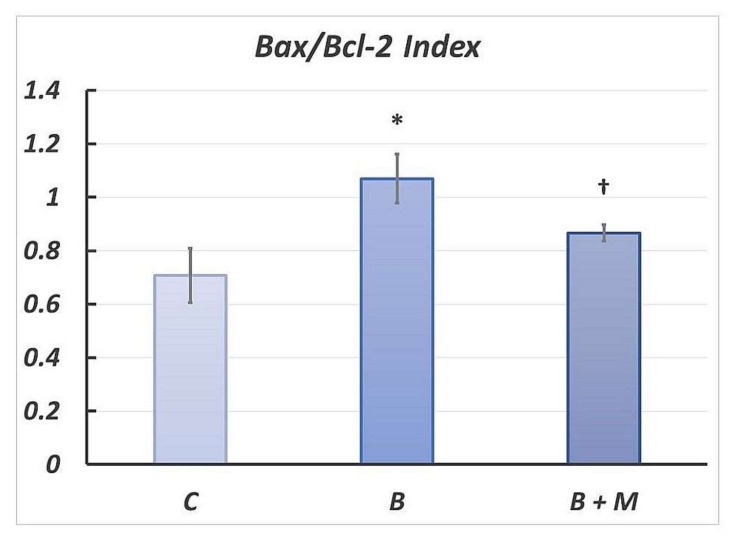
Melatonin effect on the Bax/Bcl-2 ratio (intensity index) in gastric mucosa. Results are given as means ±SEM. * *p* < 0.05 vs. control group; ^†^ *p* < 0.05 vs. burned, non-treated group. Controls (C); burned rats (B); burned melatonin-treated rats (B + M).

The Bax/Bcl-2 index was statistically significantly increased by 51% (*p* < 0.05) in the burned group compared to the control one. After melatonin treatment, the Bax/Bcl-2 index was statistically significantly reduced by 19% (*p* < 0.05) when compared to the burn group with a tendency to approach that of the control one (Figure 4).

### 2.5. The Melatonin Effect on Nrf2 Expression in Gastric Mucosa

**Figure 5:**
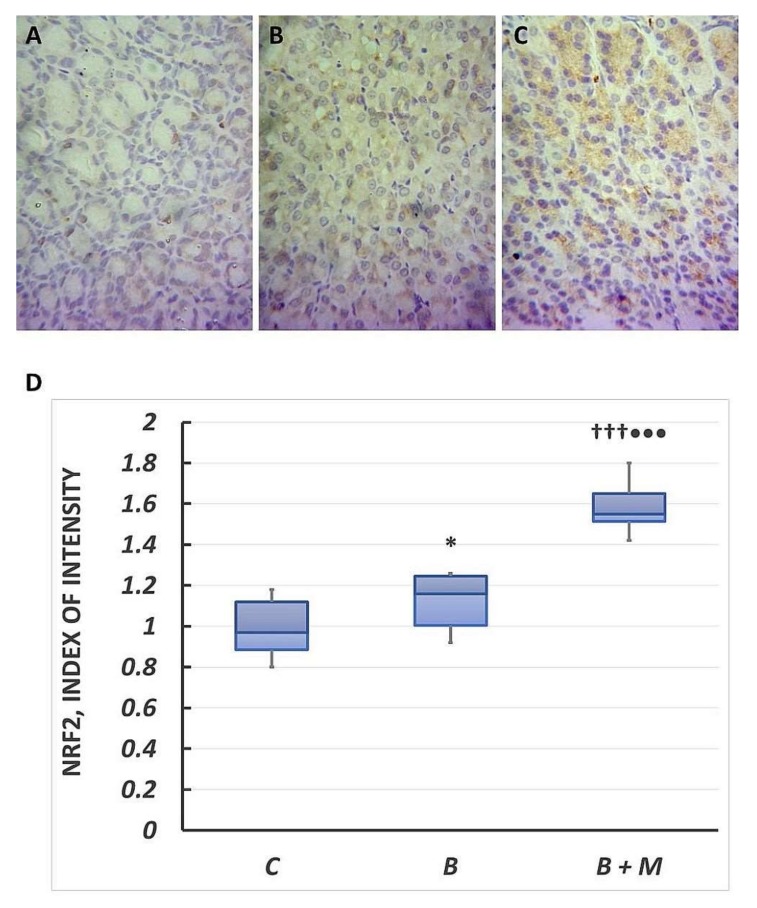
Melatonin effect on Nrf2 protein expression. Immunohistochemical Nrf2 detection in gastric mucosa. Controls (**A**); burned rats (**B**); burned rats, treated with melatonin (**C**). The antigen site appears as a brown color. Representative images. Original magnification, 400×. Score index of Bax positive immunostained cells (**D**). Results are given as box plot, with median, 25th- and 75th-percentile values, and min and max values. * *p* < 0.05 vs. control group; ^†††^ *p* < 0.001 vs. burned, non-treated group; ^•••^ *p* < 0.001 vs. control group. Controls (C); burned rats (B); burned melatonin-treated rats (B + M).

Immunohistochemical analysis showed transcription factor Nrf2 expression in both the cytoplasm and the nuclei of epithelial cells in the control group. The reaction intensity was weak with the cytoplasmic content of 0.97 (0.89–1.12) (Figure 5A). In contrast, the burned group was presented with predominantly cytoplasmic Nrf2 expression. The intensity of the reaction ranged from weak to moderate (1.16 (1.01–1.25)) and was significantly higher by 20% (*p* < 0.05) as compared to the control group (Figure 5B). After melatonin treatment, Nrf2 was detected in almost all gastric mucosal cells. The expression was predominantly moderate (1.55 (1.52–1.65)) and significantly higher compared to both groups (34% vs. burned group (*p* < 0.001); 60% vs. control one (*p* < 0.001)) (Figure 5C).

## 3. Discussion

Thermal trauma induces stress-related gastric injury [21,22]. The burned group showed higher 4-HNE levels and pro-apoptotic protein Bax levels, and significantly higher Nrf2 levels and Bax/Bcl-2 ratios in gastric tissue, but significantly lower anti-apoptotic protein Bcl-2 levels.

This injury is a strong inducer of oxidative stress. The excessive ROS production might overwhelm antioxidant defense system and generate high toxic lipid peroxides such as 4-HNE, which is considered the most significant lipid peroxidation product. Its high or very high levels lead to cell death [23,24].

Mitochondria are particularly sensitive to the harmful effect of lipid peroxides. The 4-HNE rapidly reacts with thiols and amino groups and modifies glutathione, mitochondrial coupling proteins, and antioxidant proteins, which induces cell death. The 4-HNE also increases both transcriptional and translational Bax expressions and stimulates protein translocation from cytosol to mitochondria. The latter increases mitochondrial outer membrane permeabilization, leads to cytochrome-C release into the cytosol, and triggers caspase activation [7,24,25].

The 4-HNE detoxification to less reactive chemical species minimizes its toxic effects on cells. Glutathione-S-transferase, aldo-keto reductases, alcohol dehydrogenases, and aldehyde dehydrogenase catalyze the three major detoxification pathways [26].

In the current study, we observed an increase in Bax protein levels and a significant decrease in Bcl-2 protein levels in the gastric mucosa of the burned group when compared to the control group. These results correspond to changes reported by other authors, who detect elevated Bax protein levels and decreased Bcl-2 protein expression after burns [27,28,29,30].

Cell fate, apoptosis, or survival depends on the balance between Bax and Bcl-2 proteins [9]. The imbalance between pro- and anti-apoptotic proteins, mediating apoptosis, has been found in several models of gastric injury induced by NSAIDs, ethanol, stress, and other factors [31,32,33].

Our results showed an elevated Bax/Bcl-2 ratio, predominantly for Bcl-2 reduction in burn group than in the control one. These changes can be associated to 4-HNE effects—the modification of essential cellular proteins, decreases in glutathione levels, and the alteration of cellular redox homeostasis [34].

Furthermore, pro-apoptotic Bax protein can be induced through the activation of death receptors, which are members of the TNF receptor gene superfamily. The ligands and corresponding death receptors include FasL/FasR, TNF-α/TNFR1, and induce Fas-associated death domain (FADD), which in turn activates caspase-8 and effector caspase-3. H. Li et al. identified that the pro-apoptotic Bcl-2 family member Bid is a linking element between the extrinsic and intrinsic pathways [35].

We have previously reported that thermal trauma increases the TNF-α levels in plasma [36]. We supposed then that TNF-α induced epithelial apoptosis via intermembrane death receptors in the experimental model. Likewise, the increased hepatocytes apoptosis was associated with an imbalance between pro-inflammatory/anti-inflammatory cytokines in burns [20]. Data suggested that TNF-α induced apoptosis of enterochromaffin-like cells by activating NF kappa B and generating NO [37,38]. In addition, immunohistochemically, we found increased iNOS expression in the epithelial cell cytoplasm in burned rats [39].

The low 4-HNE levels enhance cellular antioxidant capacity and exert adaptive response because 4-HNE acts as a signal molecule and regulates several transcription factors such as Nrf2 and NF-κB such that the cells can survive [24].

The transcription factor Nrf2 plays an important role in the regulation of apoptosis. Our results showed a slightly increased expression of Nrf2, mainly in the epithelial cell cytoplasm in animals with burns versus controls.

Nrf2 is a key factor in antioxidant protection and preservation of cell homeostasis. It is located in the cytoplasm and is suppressed under a basal condition through Keap1 repressor protein. Under stress (oxidative, electrophilic) and endogenous chemicals (NO, 4-HNE), Keap1-Cul3 ligase function is impaired and Nrf2 degradation is declined. Nrf2 accumulates in the cytoplasm and translocates into the cell nucleus. The heterodimer Nrf2- small Maf protein binds to an antioxidant response element, which is a promoter region of many genes [11,40]. Nrf2 activates genes that encode phase II detoxifying enzymes and antioxidant enzymes such as glutathione S-transferases and heme oxygenase and mediates the regulation of redox balance and antioxidant defense in cell.

Niture and Jaiswal report that Nrf2 releases Bcl-xL in mitochondria, increases in Bcl-xL heterodimerization with Bax in mitochondria, and reduces cellular apoptosis. It mediates elevation of the anti-apoptotic protein Bcl-2 [12], upregulates Bcl-2/Bax expression, and decreases expression and activity of caspases 3 and 9 [41].

On the other hand, the anti-apoptotic Nrf2 effect may be associated with increasing expression of glutathione-S-transferase, aldoketo reductases, alcohol dehydrogenases, and aldehyde dehydrogenase [11]. Significant amounts of these enzymes are found in mitochondria. They detoxificate 4-HNE to less reactive chemical species and decrease its pro-apoptotic action [26].

In our model, the melatonin-treated group, compared to the burned group significantly reduced 4-HNE levels and significantly increases Bcl-2 protein expression. There was a similar increase in the pro-apoptotic Bax protein. Moreover, melatonin significantly decreased the Bax/Bcl-2 index through the elevation of the Bcl-2 level, and further enhanced Nrf2 expression.

We think the protective action of melatonin is associated with the increase in Bcl-2 protein expression, despite the amplified increased expression of the pro-apoptotic Bax protein. Our results indicate that the Bax/Bcl-2 index reflects the state of gastric epithelial cells, apoptosis or survival, more accurately than the Bcl-2 protein increases in the burned group alone do. In support of this fact, we have previously reported the absence of vascular congestion, inflammatory infiltration, and apparent necrotic changes after melatonin treatment [42]. According to another study, melatonin decreases the Bax/Bcl-2 index and cell apoptosis in indomethacin-induced gastric mucosal injury [43].

The exact mechanisms of the gastroprotective effect of melatonin have been discussed. The beneficial effect of melatonin is associated with decreased Bax protein expression and increased expression of the Bcl-2 protein [43] as well as with a downregulated Bax/Bcl-2 ratio. Other authors proposed that the anti-apoptotic action of melatonin may by responsible at least in part for its antioxidant effect by increasing Nrf2 expression [44].

Melatonin, a functionally pleiotropic molecule, is also known as a powerful antioxidant. Due to its amphiphilic properties, this hormone freely passes into the cytosol and reaches all subcellular structures [45]. Melatonin and its metabolites (AFMK and AMK) also directly scavenge ROS/RNS [46,47]. It enhances the glutathione synthesis and the maintenance of glutathione homeostasis, which is important for the inhibition of oxidative stress and protective action of the latter on mitochondria and other subcellular structures [48,49]. Melatonin may interact with the lipid bilayer and stabilize the internal mitochondrial membrane; it may also inhibit both the permeability of mitochondrial pores and the release of Ca^2+^ and cytochrome C [50]. In addition, it improves energy production in mitochondria and restricts cellular damage and even cell death [49,51]. Moreover, newer studies show the ability of melatonin to induce a strong re-localization of Bcl-2, suggesting that Bax activation may in fact be antagonized by Bcl-2 at the mitochondrial level. Melatonin allows mitochondrial translocation of the pro-apoptotic protein Bax, but it impairs its activation/dimerization [15]. Based on this, we speculate that this is the mechanism of Bax neutralization by melatonin in our experimental model.

In addition, melatonin reduces the membrane cytotoxic activity of TNF-α [52] and overproduction of NO/iNOS/ONOO- as well as diminishes their deleterious effects on mitochondria [53]. Our data support these claims [39].

**Figure 6:**
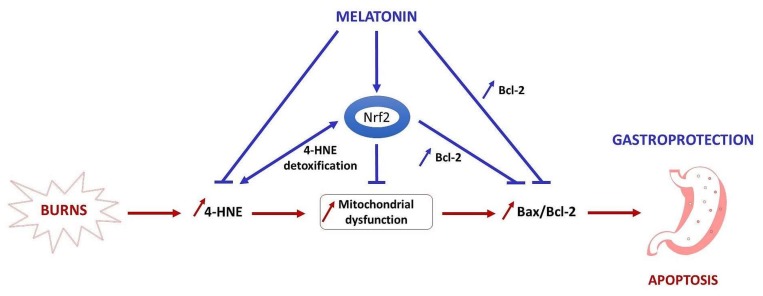
A schematic representation of molecular mechanisms and effects of melatonin in burn trauma. The experimental evidence indicates that exogenous melatonin exhibits a gastroprotective effect.

The information available about melatonin’s effect on the redox sensing transcription factor Nrf2 as a regulator of antioxidant enzymes, antioxidants, and antioxidant protection of the stomach is limited. Presumably, melatonin as electrophile increases Nrf2 expression via Nrf2/Keap1 dissociation and Nrf2 translocation to the nucleus. There are many processes in which melatonin is a key player in the complex defense network of the cell. The molecular mechanisms and effects of melatonin on gastric mucosal injury after experimental thermal trauma have thus been illustrated (Figure 6).

## 4. Materials and Methods 

### 4.1. Animals

The experimental procedure was approved by the Home Office for Care and Use of Laboratory Animals of Medical University of Varna and performed with a strong consideration for ethics of animal (90000088/2008) experimentation according to the International Guiding Principles for Animal Research approved in Bulgaria. 

Age-matched male Wistar rats weighing between 220 and 250 g, fasted for 12 h, were allowed free access to water before injury. Animals were housed in a 20 °C and offered rat chow and water ad libitum. They were kept in dark/light cycles (DL = 12:12 h) in individual wire-bottomed cages. Thus, lights were turned off at 8:00 p.m. and turned on at 8:00 a.m. to achieve a satisfactory photoperiod.

### 4.2. Thermal Injury and Melatonin Treatment

For the experimental procedure, 21 animals were randomly divided in three groups (*n* = 7 in each group) as followed: the control (C), i.e., the non-burned, non-treated group, the vehicle-treated burned group (B), and the melatonin-treated burned group (B + M). After light ether inhalation, general anesthesia was performed using thiopental (30 mg/kg i.p.). In order to accomplish a third degree burn over 30% of the total body surface area (TBSA), hot boiling water (90 °C) was applied on the back of the animals during a period of 10 s. For those rats that were subjected to burn injury, 4 mL of physiological saline were applied intraperitoneally (i.p.) for immediate resuscitation following burn injury. No animals died within the first 24 h of post-burn period. 

Either melatonin (*N*-acetyl-5-methoxytriptamine, Merck, Darmstadt, Germany) at a dose of l0 mg/kg body weight (b.w.) dissolved in vehicle or vehicle (2% ethyl alcohol diluted in physiological saline to constitute 5 mL/kg i.p.) was administered, respectively. Melatonin and vehicle were applied immediately i.p. after burns in the morning between 8:00 and 9:00 a.m. and 12 h after thermal skin injury. All animals were given buprenorphine (0.3 mg/kg i.p. b.w.) twice daily for pain control post-burn. They were re-anesthetized with thiopental and sacrificed 24 h after burns as the stomach was sampled.

### 4.3. Paraffin Processing of Tissue

Tissue specimens of gastric oxyntic mucosa were fixed in 10% buffered formalin (pH 7.2), dehydrated in an ascending series of ethyl alcohol (70–100%), and embedded in paraffin wax. Tissue sections with thicknesses of 5 μm were stained with hematoxylin and eosin (H&E) and examined using a light microscope (Olympus BH-2, Tokyo, Japan). Histopathological changes were evaluated at a magnification of 400× (a high power field).

### 4.4. Immunohistochemistry

The deparaffinized and dehydrated sections were treated with 1% hydrogen peroxide for peroxidase activity inhibition for 5 min. Then, they were rinsed in 0.1 M phosphate buffered saline (PBS) (pH 7.4) and treated with normal goat serum for 20 min. Subsequently, the sections were incubated with primary antibody for 24 h at room temperature. The following antibodies were used: 4-HNE (Abcam, Cambridge, UK) at a dilution of 1:200 and Bcl-2 (N-19), Bax (C-20), and Nrf2 (С-20) (Santa Cruz, CA, USA) at a dilution of 1:50. Finally, peroxidase activity was estimated by the diaminobenzydine-tetrachloride H_2_O_2_ method. Negative controls were incubated with non-immune sera instead of primary antibody.

A morphometric method was used to assess quantitatively the contents of 4-HNE, Bax, Bcl-2, and Nrf2. The content was determined as strong, score 3, moderate, score 2, weak, score 1, and lacking, score 0, on the basis of the occurrence of immunodeposits (Tzaneva, 2001) [54]. The 4-HNE, Bax, Bcl-2, and Nrf2 concentrations of the epithelial cells were defined as the content of each cell multiplied by their scoring factors, which was divided by the total number of cells ((content x scoring factor)/total number of cells). Morphometric investigation was performed on 50 cells from each sample. Three blinded observers counted immuno-positive cells and the data were pooled.

### 4.5. Statistical Analysis

GraphPad Prism 6.0 was used for statistical analysis. Data were presented as median and interquartile range (IQR) (25th–75th percentile) and mean ±SEM. The statistical significance of difference was evaluated with the Mann–Whitney U test and a Student’s unpaired t-test. A P-value less than 0.05 was considered to indicate statistical significance.

## 5. Conclusions

Thermal trauma induces gastric mucosal oxidative injury. Melatonin limits lipid peroxidation and 4-HNE accumulation, and ameliorates burn-induced gastric mucosal injury and apoptosis. We found that melatonin gastroprotection is accomplished via activation of the transcription factor Nrf2, modulation of Bcl-2 family protein the expression and reduction of Bax/Bcl-2 ratios in experimental thermal trauma. This is the first report to show that melatonin activates the Nrf2/signaling pathway and activation of apoptotic network in gastric mucosa after experimental burn injury. 

Further studies on the activation of Nrf2 and the regulation of Bcl-2 family proteins are needed to clarify the gastroprotective effect of melatonin in experimental burns.
